# Diet Quality and Its Relationship with Weight Characteristics in Pregnant Japanese Women: A Single-Center Birth Cohort Study

**DOI:** 10.3390/nu15081827

**Published:** 2023-04-10

**Authors:** Chihiro Imai, Hidemi Takimoto, Kayo Kurotani, Ayako Fudono, Iori Tarui, Tomoko Aoyama, Satoshi Yago, Motoko Okamitsu, Naoyuki Miyasaka, Noriko Sato

**Affiliations:** 1 Faculty of Education, Graduate Faculty of Interdisciplinary Research, University of Yamanashi, 4-4-37 Takeda, Kofu 400-8510, Japan; 2 Department of Nutritional Epidemiology and Shokuiku, National Institutes of Biomedical Innovation, Health and Nutrition, NK Building, Kento Innovation Park, 3-17 Shinmachi, Senrioka, Settsu 566-0002, Japan; 3 Faculty of Food and Health Sciences, Showa Women’s University, 1-7-57 Taishido, Setagaya-ku, Tokyo 154-8533, Japan; 4 Comprehensive Reproductive Medicine, Graduate School of Medical and Dental Sciences, Tokyo Medical and Dental University, 1-5-45 Yushima, Bunkyo-ku, Tokyo 113-8510, Japan; 5 Child and Family Nursing, Graduate School of Health Care Sciences, Tokyo Medical and Dental University, 1-5-45 Yushima, Bunkyo-ku, Tokyo 113-8510, Japan; 6 Department of Food and Nutrition, Faculty of Human Sciences and Design, Japan Women’s University, 2-8-1 Mejirodai, Bunkyo-ku, Tokyo 112-8681, Japan

**Keywords:** nutrient rich food index 9.3 (NRF9.3), japanese food guide spinning top (JFGST), pre-pregnancy body mass index (BMI), gestational weight gain (GWG)

## Abstract

Maternal underweight and inadequate gestational weight gain (GWG) are problems in Japan. However, increases in food intake aimed at weight gain alone are not sufficient for mother–child health. This study assessed diet quality based on the 3-day dietary records of pregnant women in an urban area of Japan in order to show the importance of evaluating diet quality, using the Nutrient-Rich Food Index 9.3 (NRF9.3), which is one metric based on nutrition profiling, and the Japanese Food Guide Spinning Top (JFGST). After excluding misreporters of energy intake, we stratified women (*n* = 91) by pre-pregnancy body mass index (BMI) and determined energy intake, diet quality status, and their relationship with GWG. Intakes of carbohydrate-containing staple foods, vegetable dishes, and fruit were insufficient regardless of BMI. Most of the underweight women with inadequate GWG had insufficient energy intake but high diet quality, as assessed by NRF9.3. In contrast, most women who consumed energy within the recommended range had low diet quality and gained weight at inappropriate levels. These results highlight the importance for pregnant Japanese women to maintain diet quality through a nutrient-dense diet, while simultaneously increasing energy intake after evaluation of their individual diet quality.

## 1. Introduction

Maternal underweight is associated with low birth weight and preterm birth. The high percentage of women with pre-pregnancy underweight and inadequate gestational weight gain (GWG) is a serious problem in Japan [1,2,3]. The Japanese Society of Obstetrics and Gynecology (JSOG) revised its GWG guidelines in 2021 [4]. Subsequently, the GWG growth curves for optimizing pregnancy outcomes were created in a manner specific to pre-pregnancy body mass index (BMI) [5]. Underweight women are now encouraged to gain as much weight as is defined in the optimal range that is specific to low BMI. Currently, nutritional advice for pregnant women in Japan encourages adherence to food-based dietary guidelines to achieve the recommended energy intake with food and nutritional balance, as shown in the Japanese Food Guide Spinning Top (JHGST) [6]. For women to achieve an ideal diet, more detailed (attentive) advice on the quantity and quality of food is needed. At present, however, little is known about the quality of the diet that is followed by underweight women in Japan.

Although there are no universal indices specific to assessing the diet quality of pregnant women, the maternal diet quality has been recently assessed abroad, where obesity is a growing problem, by using indices such as the Healthy Eating Index (HEI), alternate HEI (AHEI), alternate Mediterranean diet (AMED), and Dietary Approaches to Stop Hypertension (DASH) [7,8,9,10,11]. Poor diet quality is often due to a high proportion of energy intake from high energy density foods such as sweets and high-fat foods. It has been shown that poor diet quality is associated with obesity in pregnant women, which further adversely affects the health of the child. Thus, the general recommendation is to reduce the energy density in order to decrease the weight as much as possible. However, this cannot be directly applied to Japan, a country where there are many underweight pregnant women. In Japan, using cluster analysis, Ohkubo et al. classified dietary patterns into three groups based on the food group intake patterns during pregnancy, and identified a group with a high dietary intake of rice, fish, vegetables, and fruit. This group exhibited, with significance, the lowest prevalence of inadequate intake of fifteen nutrients, representing the best profile in terms of nutritional adequacy for the dietary reference intake (DRI) [12]. This group had moderate energy intake levels and showed similar pre-pregnancy BMI levels to other groups but the lowest GWG. Nevertheless, this group showed the highest birth weight among the three groups [13]. Thus, the relationship between nutritional status and weight characteristics is not straightforward in Japan.

We have previously shown that the Nutrient-Rich Food Index 9.3 (NRF9.3), a validated diet quality index that is well correlated with the HEI [14], can be used to assess the overall diet quality of pregnant Japanese women [15]. NRF9.3 is a qualified nutrient profiling system developed by Prof. Drewnowski according to the European Food Safety Authority Guidelines [16]. Because the NRF9.3 is a nutrient-based assessment, it universally evaluates diet as well as foods. In the present study, we assessed the overall diet quality by NRF9.3 and intake levels of energy and five food groups classified by JHGST across pre-pregnancy BMI categories and determined their relationship with GWG in order to show the importance of evaluating diet quality when providing nutritional support during pregnancy.

## 2. Materials and Methods

### 2.1. Study Population

The present research was based on a prospective mother-child cohort study in the Metropolitan Tokyo area: the Birth Cohort Gene and ENvironment Interaction Study of Tokyo Medical and Dental University (TMDU) (BC-GENIST), as previously described [15,17,18,19]. Participant recruitment took place from 2015 to 2019. The study was approved by the Ethics Review Committee of the Faculty of Medicine and the Medical Research Institute of TMDU (G2000-181). A complete dataset for the nutritional survey and weight characteristics were available for 108 out of 126 participants with singleton pregnancies. Of these, 91 participants who were acceptable reporters of energy intake were considered eligible for this study. The determination of the adequacy of energy intake reporting has been described in detail in a previous study [15]. In brief, the Goldberg cutoff method [20,21] was used to identify and exclude those who reported implausible energy intake.

### 2.2. Data Collection

In this cohort study, non-consecutive 3-day dietary records were obtained from the study participants during the mid-gestation period (the median value [interquartile range] of the period for data collection was 19 weeks; range, 17–22 weeks), as previously described [15]. Data on maternal age, height, weight, parity, fetal sex, and birth weight were collected from the medical records. GWG was calculated as the difference between maternal weight at 40 weeks of gestation and pre-pregnancy weight. Maternal weight at 40 weeks was obtained via an observed clinical measurement. In other cases when the mother did not deliver at 40 weeks, a predicted value of the GWG at 40 weeks was calculated from the rate of weight gain, as previously described [22]. Birth weight was adjusted for gestational age, fetal sex, and parity using a reference chart [23] and then scaled by Z score transformation, as previously described [18]. A preliminary study was conducted on a subset of the participants to determine whether the habitual physical activity levels (before pregnancy) are related to pre-pregnancy BMI. We investigated the daily total physical activity level using a self-administered questionnaire called the Shorter Version of the Physical Activity Questionnaire [24]. This questionnaire has been validated to rank subjects relative to the level of their physical activity, although it is insufficient in terms of quantitatively predicting physical activity. Since the results showed that physical activity level did not affect BMI (Figure S1), we did not include a parameter of physical activity level in the present study.

### 2.3. Dietary Assessment

Dietary intake was assessed from the 3-day food dietary records. A detailed description of the procedure has been previously published [15]. In brief, upon collection of the food dietary records, all the recorded sheets were checked by dietitians to clarify any ambiguous points. The dieticians then converted the portions of the foods consumed into estimated intake (g). When a raw food such as vegetables, meat, fish, or eggs was cooked and not eaten raw, the method of cooking was also checked and recorded. Each food item was coded according to the food numbers in the Standard Tables of Food Composition (STFC) in Japan, 2015 [25], so that the energy and nutrient intake could be calculated.

### 2.4. NRF9.3 and Assessing the Stability of NRF9.3 Ranking against Day-to-Day Food-Intake Variation

NRF9.3 is a composite index of nutrient density that can be utilized for the assessment of an individual’s overall diet quality [26]. In brief, the NRF9.3 score was calculated as the sum of the percentage of reference daily values (RDV) for nine qualifying nutrients (protein, dietary fiber, vitamin A, vitamin C, vitamin D, calcium, iron, potassium, and magnesium) minus the sum of the percentage excess of RDV for three disqualifying nutrients (added sugars, saturated fatty acids, and sodium). Age-specific RDVs for pregnant women at mid-gestation were determined based on the Dietary Reference Intakes (DRIs) for the Japanese population, 2015 [27]. We recruited the participants during 2015–2019 and started to investigate nutrient/food intake and diet quality prior to 2020 using the 2015 STFC and the 2015 DRI for Japanese population. For consistency in our analyses, we used the same version of the DRI throughout the study. We previously calculated the NRF9.3 score based on the mean values of the nutrient intake over three days in order to assess the overall diet quality of the participants [15].

Some of the components of the NRF9.3, such as vitamins, are known to exhibit a large day-to-day (daily) variation in the intake. Therefore, we evaluated the influence of the day-to-day fluctuations in ranking on the NRF9.3 through the use of the following procedure. First, the NRF9.3 for each day was calculated separately for the 1st, 2nd, and 3rd day of the dietary record for each participant. Next, we randomly selected one NRF9.3 score for any of the three days for each participant. Subsequently, we then ranked all of the participants according to NRF9.3 and stratified them by tertiles. The participants were categorized as Low_day (*n* = 30), Intermediate_day (*n* = 30), and High_day (*n* = 31), which covered the scores from the lowest to the highest NRF9.3 scores. These series of operations were performed 1000 times and then used to determine the probability of each participant being classified as Low_day, Intermediate_day, or High_day. Independently, tertile classification in accordance with the NRF9.3 score was performed based on the mean value of the nutrient intake over a 3-day period, and the participants were categorized as Low_period (*n* = 30), Intermediate_period (*n* = 30), and High_period (*n* = 31). To evaluate the stability of the NRF9.3 ranking, the mean of the probabilities of the Low_day of the Low_period members was calculated. Similarly, we also calculated the mean of the probabilities of the Intermediate_day and the High_day of the Intermediate_period and High_period members, respectively.

### 2.5. Japanese Food Guide Spinning Top (JFGST) for Pregnant Women

The JFGST was created as a tool to help Japanese people to understand food-based dietary guidelines and to practice healthy eating [28]. The guidelines encouraged well-balanced meals consisting of (1) staple foods containing carbohydrates, (2) side dishes containing vegetables and/or other foods, (3) main dishes containing protein, (4) milk products, and (5) fruit. One serving (SV) unit for each dish corresponded to 40 g of carbohydrate for staple foods (also called grain dishes), 70 g of uncooked vegetables/potatoes/seaweeds/mushrooms for side dishes (vegetable dishes), 6 g of protein from fish/meat/eggs/soy products for main dishes (fish and meat dishes), 100 mg of calcium for milk, and 100 g of fruit for the various fruits. In the case of 100% vegetable juice or 100% fruit juice, half the weight of the amount consumed was counted. The guidelines also provided the recommended daily SV numbers for the above five components and the recommended daily intake of the total energy and energy from snacks and beverages for adults [6]. For pregnant women, the Dietary Guidelines for Pre-pregnant, Pregnant, and Lactating Women in Japan 2021 [1] provided the additional SV numbers specific to each gestational period. We used the recommended daily standards for pregnant women at mid-gestation: 5–7 SVs for staple dishes (grain), 6–7 SVs for side dishes (vegetables), 4–6 SVs for main dishes (fish and meat), 2 SVs for milk, 3 SVs for fruit, 2000–2400 kcal for total energy, and 0–200 kcal for energy from snacks and beverages.

To measure adherence to the Japanese Food Guide, the JFGST adherence scores were calculated as described in a previous study [29]. In brief, each JFGST component score is defined as a maximum score of 10 and a minimum score of 0. The highest score is given only if the intake is within the recommended range, with scores deducted according to the percentage of deficiency or excess. For the side dishes and fruit, the upper limits were eliminated in accordance with the methods of a previous study [29]. Table S1 describes the calculation method for the JFGST score.

### 2.6. Adequacy of GWG by Pre-Pregnancy BMI

Recently, a large-scale epidemiological study determined the appropriate range of GWG specific to the pre-pregnancy BMI for pregnant Japanese women [5]. This range was determined on the basis of an analysis of the GWG patterns in a low-risk population of 17950 women with singleton pregnancies without any complications during the pregnancy through one-month postpartum. The range of GWG for each pre-pregnancy BMI category (underweight, <18.5; normal weight, 18.5–24.9; overweight, 25–29.9 kg/m^2^) was 12–15 kg, 9.9–12.8 kg, and 7–10 kg for underweight, normal weight, and overweight, respectively [5]. We compared the participants’ GWGs to these appropriate GWG ranges based on an analysis of a low-risk population to assess the adequacy of the GWGs. As shown in the Supplementary Material (Table S2), we classified the participants whose GWG was within the appropriate GWG range specific to the corresponding BMI as adequate, below the range as inadequate, and above the range as excessive. Of note, the appropriate GWG range based on this low-risk population analysis was almost the same as that for the JSOG’s guideline for GWG (12–15 kg, 10–13 kg, and 7–10 kg for underweight, normal weight, and overweight, respectively).

### 2.7. Statistical Analysis

R software (version 4.0.3) and SPSS statistical software (version 24; IBM Corp., Armonk, NY, USA) were used for the statistical analyses.

Descriptive statistics of maternal demographic characteristics were generated according to the general classification of underweight, normal weight, and overweight. With this classification, the number of participants in the overweight group was very small. To analyze the relationship between maternal characteristics and BMI, linear or logistic regression analyses for the entire population were performed, using BMI as a continuous variable.

A linear regression model was used to examine the association of dietary intake and overall diet quality with log-transformed (due to the skewness of the BMI distribution) pre-pregnancy BMI as a continuous variable. Covariates were variables previously reported to affect the food intake of pregnant women (maternal age [continuous], educational attainment [dichotomous categorical variable], and household income [dichotomous categorical variable]) [13,29,30]. When the SV numbers of milk and fruit and energy intake from snacks were used as dependent variables, they were Box–Cox transformed.

To examine the association of GWG and birth weight in conjunction with the overall diet quality as a continuous variable, a linear regression model was used. Covariates were variables previously reported to affect GWG and birth weight (pre-pregnancy BMI [continuous, log-transformed], maternal age [continuous], educational attainment [dichotomous categorical variable], and household income [dichotomous categorical variable]).

All reported *p*-values were two-tailed, and *p*-values < 0.05 were considered statistically significant.

## 3. Results

### 3.1. Characteristics of Participants According to Pre-Pregnancy BMI

Maternal characteristics according to pre-pregnancy BMI category are shown in Table 1. Approximately 20% of the participants were underweight based on the pre-pregnancy BMI. Regression analyses showed that maternal age, height, and other characteristics were not significantly associated with pre-pregnancy BMI (Table 2).

### 3.2. Assessing the Stability of Diet Quality Classification Using NRF9.3 Ranking Based on 3-Day Dietary Records

The classification of individual diet quality based on the mean value of the 3-day dietary records was verified (Table 3). Members who were ranked as high or low diet quality according to the 3-day dietary records were ranked even by any single day record as the same quality levels with high probability: 0.70 for high and 0.74 for low diet quality. In contrast, the probability of classification discordance between the classification by 3-days and that by any single day was quite low: 0.034 for high and 0.069 for low diet quality. From this, it was confirmed that the classification of the diet quality by the 3-day meal records was reproducible.

### 3.3. Diet Quality and Quantity According to Pre-Pregnancy BMI

**Figure 1:**
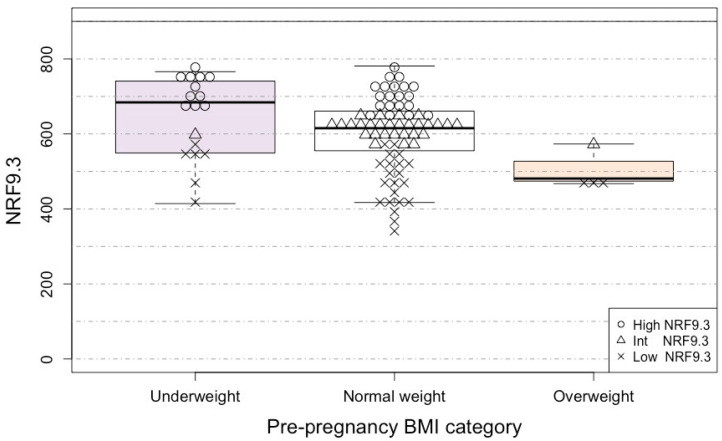
NRF9.3 by pre-pregnancy BMI category. Each subject (*n* = 91) is shown in the box-and-whisker plots, with the boxes indicating the median and interquartile range and the whiskers denoting the range. Higher NRF9.3 scores indicate better overall diet quality. A maximum possible NRF9.3 score is 900 (solid line). Light purple, white, and pink indicate underweight, normal weight and overweight. Open circles, open triangles, and crosses indicate high, intermediate, and low NRF9.3 scores, respectively. NRF9.3, Nutrient Rich-Food Index 9.3; BMI, body mass index.

Analysis of NRF9.3 by pre-pregnancy BMI category showed that it was higher in underweight individuals and lower in overweight individuals (Figure 1). The scores of the NRF9.3 tertile group were 333 ≤ Low NRF9.3 ≤ 570; 573 ≤ Intermediate NRF9.3 ≤ 653; and 655 ≤ High NRF9.3 ≤ 781. In Figure 1 and the following figures in the main text, the participants with low, intermediate, and high NRF9.3 scores are presented as crosses (×), triangles (△), and circles (○).

**Figure 2:**
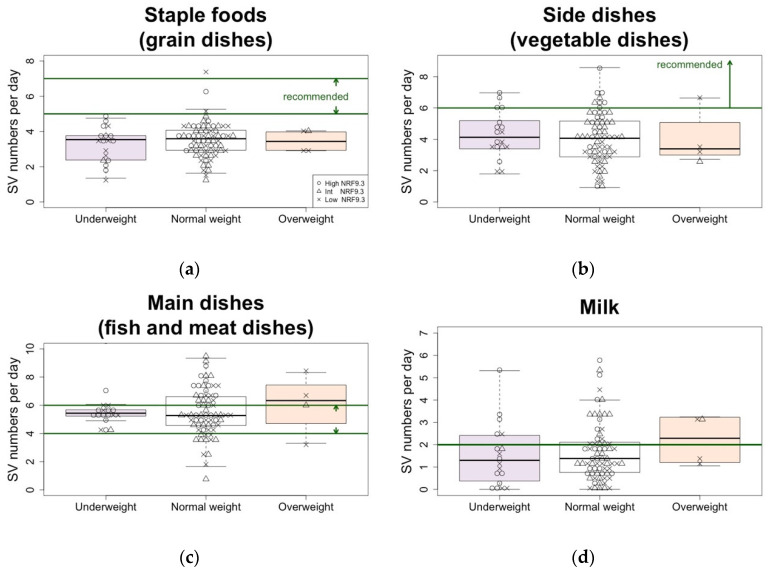

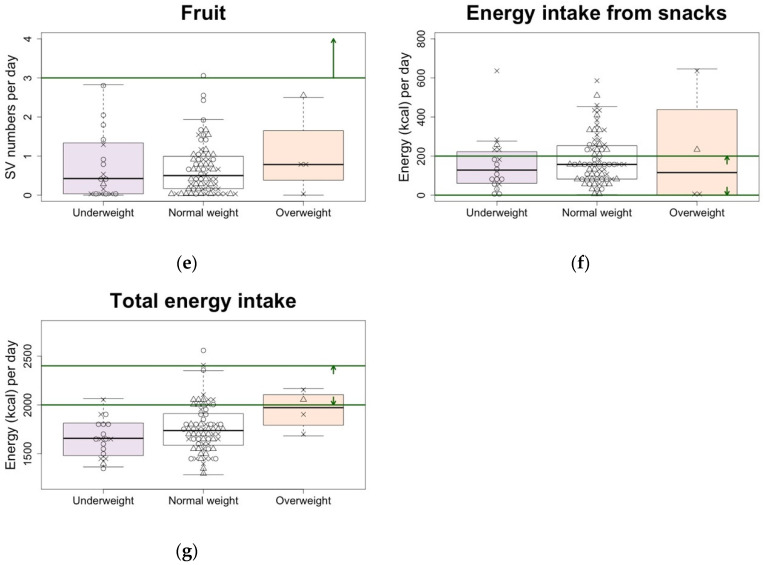
JFGST SV number (**a**–**e**) and energy intake (**f**,**g**) according to the pre-pregnancy BMI category, with presentation of the individual’s diet quality status. Each subject (*n* = 91) is shown in the box-and-whisker plots, with the boxes indicating the median and interquartile range and the whiskers denoting the range. Green solid line indicates the recommended SV number or its recommended range. Light purple, white, and pink indicate underweight, normal weight, and overweight. Open circles, open triangles, and crosses indicate high, intermediate, and low NRF9.3 scores, respectively, in order to show the relation between food intake and overall diet quality. JFGST, Japanese Food Guide Spinning Top; SV, serving; NRF9.3, Nutrient Rich-Food Index 9.3; BMI, body mass index.

Regarding each JFGST component, the intake of staple foods (grain dishes), side dishes (vegetable dishes), and fruit were below the recommended range, regardless of the pre-pregnancy BMI (Figure 2). 

In addition to the number of SVs, we evaluated the degree of adherence to the Japanese dietary guidelines by the JFGST adherence score. There was no difference in any BMI category (Table S3). However, there were differences in the adherence scores among the components. Scores for the fruit component were particularly low, with the underweight and normal weight groups scoring less than 3 out of 10.

We further examined the relationship between the pre-pregnancy BMI as a continuous variable and NRF9.3 after adjusting for covariates such as maternal age, educational attainment, and household income (Table 4). NRF9.3 was negatively associated with the pre-pregnancy BMI (β = −280, *p* = 0.0032). Similarly, we examined the relationship between the pre-pregnancy BMI and JFGST SV number or energy intake. Although total energy intake was positively associated with the pre-pregnancy BMI (β = 549, *p* = 0.019), there was no association between the intake of any other JFGST component and the pre-pregnancy BMI. Although NRF9.3 and JFGST score were correlated (r = 0.57, *p* = 3.5 × 10^−9^), there was no association found between the JFGST score and the pre-pregnancy BMI (Table 4).

### 3.4. Relationship among Diet Quality, Energy Intake, and GWG Adequacy

GWG was negatively associated with higher diet quality after adjusting for maternal age, pre-pregnancy BMI, educational attainment, and household income (*p* for trend = 0.009) (Table 5). However, there was no difference observed for the GWG among the JFGST score tertiles (T1, 10.3 ± 3.8; T2, 10.4 ± 2.6; T3, 9.9 ± 3.8; *p* for trend = 0.937). 

In general, reduced GWG decreases birth weight. However, even though high NRF9.3 tended to reduce GWG, it did not decrease the birth weight (Table 5). Neither GWG nor birth weight was associated with energy intake (Table S4).

**Figure 3:**
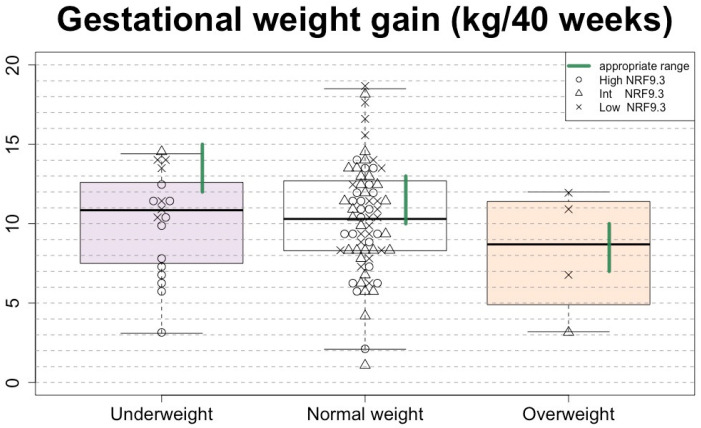
Gestational weight gain (GWG) according to the pre-pregnancy BMI category, with presentation of the individual’s diet quality. Each subject (*n* = 91) is shown in the box-and-whisker plots, with the boxes indicating the median and interquartile range and the whiskers denoting the range. Open circles, open triangles, and crosses indicate high, intermediate, and low NRF9.3 scores, respectively. Green lines indicate the BMI-specific optimal GWG ranges (12–15 kg, 9.9–12.8 kg, and 7–10 kg for underweight, normal weight, and overweight) determined by [5].

The optimal range of GWG (shown as the green line in Figure 3) depends on pre-pregnancy BMI. Inadequate GWG (below the green lines) accounted for 50% of the total. Adequate and excessive GWG accounted for 30% and 20%, respectively. Moreover, 72% of underweight women showed inadequate GWG (Figure 3). Of note, most underweight women with adequate GWG had low NRF9.3 scores (Figure 3). The overweight women showed either inadequate or excessive, but not adequate, GWG, and all had low NRF9.3 scores (Figure 3).

**Figure 4:**
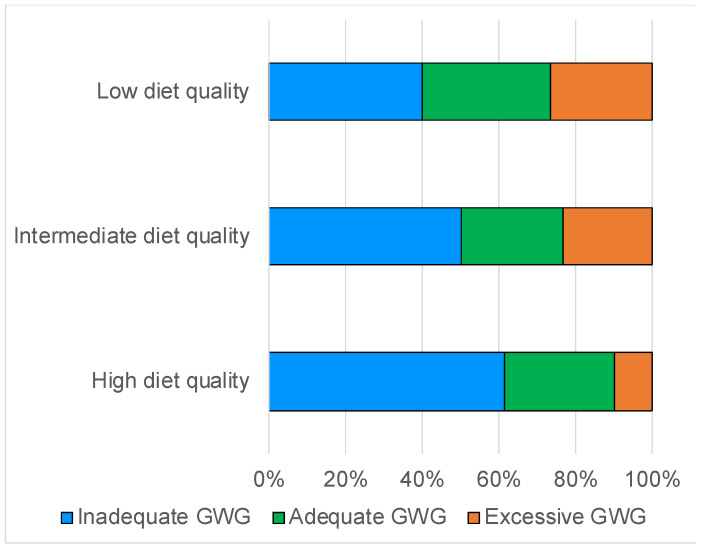
Relationship between diet quality assessed by NRF9.3 and GWG adequacy. Proportions (%) of inadequate, adequate, and excessive GWG across NRF9.3 are shown. Low, intermediate, and high diet quality correspond to low, intermediate, and high NRF9.3 score group, respectively.

The high quality of the diet decreased the excessive GWG but did not increase the proportion of adequate GWG (Figure 4).

In contrast, the high JFGST scores increased the percentage of excessive GWG (Figure S2).

Figure S3 scatter plots show the relationship between NRF9.3 and JFGST in the underweight (a) or normal weight (b) groups, color-coded with GWG adequacy presentation (blue, inadequate GWG; green, adequate GWG; orange, excessive GWG). Many of those who had both high JFGST and NRF9.3 (high diet quality) had inadequate GWG, especially in the underweight group.

**Figure 5:**
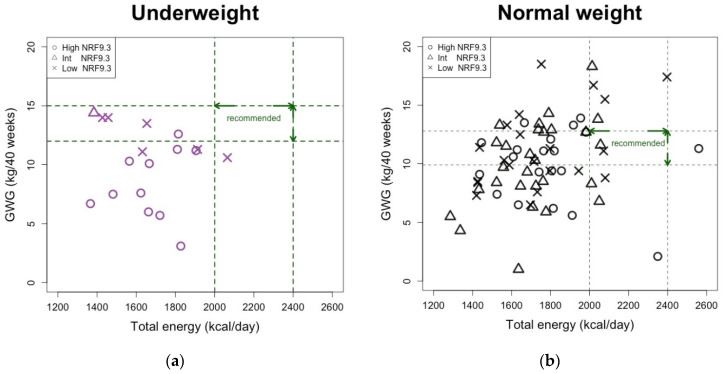
Relationship between energy intake and GWG, with presentation of the individual’s diet quality status in (**a**) underweight and (**b**) normal weight women. Each subject ((**a**), *n* = 18; (**b**), *n* = 69) is shown in a scatter plot. Open circles, open triangles, and cross indicate high, intermediate, and low NRF9.3 scores, respectively. Green dashed lines indicate the recommended range.

Furthermore, higher energy intake did not simply result in higher GWG, especially in underweight women. There was no correlation between total energy intake and GWG (r = −0.14, *p* = 0.59). Even when GWG was in an adequate range, both the energy intake and diet quality tended to be low (Figure 5a). In normal weight, GWG tended to increase as energy intake increased (r = 0.26, *p* = 0.030). Even at normal weight, most women did not meet the recommended energy intake levels, but even if they met sufficient levels of energy intake, most of them were neither in the appropriate GWG range nor in the high diet quality (Figure 5b).

## 4. Discussion

This study investigated the quality and quantity of dietary intake using the NRF9.3 and JFGST adherence scores of pregnant Japanese women according to their pre-pregnancy BMI category and examined the relationship with their GWG adequacy. We found that (1)nergy intake was inadequate in underweight and normal weight groups; (2) inadequate intake of three JFGST components (staple foods, vegetables, and fruit) was observed in all BMI categories; (3) most of the underweight and GWG-inadequate women had high diet quality as assessed by NRF9.3; (4) none of the overweight women achieved adequate GWG and all had low diet quality; and (5) women who consumed energy in the recommended range had lower diet quality and were less likely to fall into the adequate GWG.

As BMI is a general surrogate marker of nutritional status, Japanese women with low pre-pregnancy BMI are considered undernourished. Indeed, consumption of faulty diets by pregnant women in Japan and their poor eating habits due to a desire to be slim have been reported [31,32]. To improve this situation, dietary guidelines for pre-pregnant, pregnant, and lactating women in Japan have been recently updated to encourage women to intake well-balanced foods, with these guidelines also setting the recommended SV number of JFGST for every stage of pregnancy [1]. Simultaneously, the JSOG revised its GWG guidelines, recommending a higher weight gain during pregnancy than the previous target [4]. While body weight can be easily measured, there are concerns that nutritional advice that focuses on weight only may actually lead to poor diet quality. Therefore, the present study clarified the relationship between weight characteristics and diet quality in pregnant women. We provide information that can be helpful for future nutritional advice to underweight pregnant women.

First, our present results showed that the pre-pregnancy BMI was negatively associated with diet quality as assessed by the NRF9.3. This result was consistent with multiple previous studies that were performed abroad [7,8,9,33,34]. In addition, pre-pregnancy BMI was positively associated with total energy intake, which is one of the JFGST components. This result was not surprising since reducing energy density and increasing nutrient density generally improve diet quality and can have a weight loss effect. Additionally, analysis of the JGFST SV number showed that most participants across all of the BMI categories were not deficient in fish and meat, dairy, and snacks but were deficient in grains, vegetables, and fruit. Neither the SV number of each dish nor the JFGST adherence score was associated with the pre-pregnancy BMI. Thus, the NRF9.3 can provide additional important information over the JGFST in assessing the diet quality of pregnant women. It also suggested that pregnant women with underweight in Japan are not necessarily undernourished.

Second, diet quality as assessed by the NRF9.3 was negatively associated with GWG but not offspring birth weight. This result was almost the same as that for a previous report in Japan that reported the group with the highest diet quality consumed diets rich in rice, fish, vegetables, and fruit and showed the least GWG but the highest offspring birth weight [13]. We further investigated the relationship between diet quality and GWG adequacy. Notably, more than half of the women with low diet quality showed adequate or excessive GWG, whereas more than half of the women with high diet quality showed inadequate GWG. Unfortunately, underweight women with adequate GWG had low energy intake and NRF9.3 scores. Many underweight women had high NRF9.3 scores but inadequate GWG. This current situation suggests that, as we noted above, promoting an increase in energy intake to gain weight may result in reduced diet quality, even if the GWG is adequate as a result.

These results indicate the difficulty of providing nutritional advice in compliance with the GWG guidelines. The insufficient establishment of guidelines for maternal diet, nutrition, and appropriate GWG has become an international problem, where it has been noted that there are limitations in ensuring adherence to the GWG guidelines [35,36]. Furthermore, being underweight in general is rather protective against pregnancy complications. Recently, the importance of more tailored approaches for ensuring a healthy diet has been recognized [35]. It is expected that nutritional management alone, or in combination with physical exercise, appropriate diet timing, individual problem solving, etc., will eventually help to effectively control GWG. In the past, advice for following a healthy diet has been promoted in Japan using the JGFST. This study showed that using metrics such as the NRF9.3 may be more appropriate for assessing the diet quality of pregnant women.

The main strength of the present study is that it shows the relationship between weight characteristics and diet quality assessed by the NRF9.3 in pregnant Japanese women. The NRF9.3 is one method of nutritional profiling that evaluates to what degree the recommended nutrients are consumed relative to the reference values and to what degree the disqualified nutrients are consumed at amounts over the upper limit. Within the same food group, some foods are of good quality while others are not, with the NRF9.3 able to capture these differences as well. Thus, since the NRF9.3 can assess diet quality in more detail than the JGFST, the NRF9.3 was used to properly evaluate the diet quality of pregnant women in the present study. We also carefully conducted the analysis in terms of the following two aspects: (1) participants who misreported their dietary intake were excluded and (2) it was verified that the diet quality classification using NRF9.3 based on 3-day dietary records was reproducible and reliable.

The present study also has several limitations. The first is related to the heterogeneity in the underweight group. We were aware that the underweight group consisted mostly of individuals scoring highly for the NRF9.3, even though the distribution of NRF9.3 scores was bimodal and included different subgroups with low NRF9.3 and high NRF9.3 scores. However, we found no differences between these subgroups for any maternal characteristic variable we could examine, including that of educational attainment. Generally, however, Japanese underweight women comprise people with various characteristics, and the relationship between these characteristics, nutrition, GWG, and child health will need to be clarified in the future. The second limitation is the population validity. This study was a small-scale single-center birth cohort study conducted in an urban area of Japan. Although we have already confirmed [15] that the weight characteristics and nutrition status were similar to those in the national survey, our cohort might not be the representative of all pregnant Japanese women. Therefore, it is likely that it will be necessary to conduct a similar study on a larger scale in Japan in the future. Third, there is the lack of well-established diet quality metrics specifically tailored to pregnant Japanese women. Currently, there is no diet quality index that has been specifically designed for pregnant women. However, since the NRF9.3 can be calculated using the Dietary Reference Intake Standards for Japanese Pregnant Women [27,37] as reference values, we believe that our present study was a proper evaluation of the diet quality for pregnant women. It should also be noted that our study does not indicate that the NRF9.3 is the only metric of diet quality for pregnant women, but rather highlights the importance of individualized assessment of diet quality using an index such as the NRF9.3 in order to provide nutritional advice. Finally, in order to fully apply the results of this research in practical terms, we believe that it is necessary for anyone to know their diet quality. Our research is currently expanding into enhancements to smartphone applications by adding the ability to calculate and provide diet quality score, with the future goal of contributing to the comprehensive improvement of maternal nutrition, including physical activity levels and life rhythms.

## 5. Conclusions

In Japan, a significant percentage of pregnant women are underweight, and they are often considered undernourished. The present study employed one established metric, the Nutrient Rich-Food Index 9.3, to assess the diet quality of pregnant women and to determine the relationship between diet quality and weight characteristics for the first time. Most of the underweight women with inadequate GWG had insufficient energy intake but high diet quality. In contrast, most women who consumed energy within the recommended range had low diet quality. The results highlighted the importance of assessing individual diet quality using appropriate metrics to prevent the deterioration of diet quality that may inadvertently occur when pursuing weight gain, especially in low-BMI pregnant women.

## Figures and Tables

**Table 1:** Maternal characteristics according to pre-pregnancy BMI category of BC-GENIST (2015–2019) participants with no misreported energy intake.

Characteristics	Underweight BMI < 18.5	Normal Weight 18.5 ≤ BMI < 25	Overweight 25 ≤ BMI
	(*n* = 18)	(*n* = 69)	(*n* = 4)
Maternal age (years)	32.3 ± 3.3	34.1 ± 4.3	32.3 ± 3.0
Height (cm)	159 ± 4.7	158.7 ± 5.7	155.1 ± 2.3
Gestational weight gain (kg/40 weeks)	10.1 ± 3.3	10.3 ± 3.4	8.2 ± 4.0
Parity (multipara)	7 (38.9)	37 (53.6)	3 (75.0)
Maternal educational attainment, university or higher degree	14 (77.8)	54 (78.3)	2 (50.0)
Household income (≥6 million yen per year)	15 (83.3)	48 (69.6)	1 (25.0)
Gestational week at delivery (week)	39.5 ± 1.3	39.1 ± 1.4	39.3 ± 2.5

Values are represented as mean ± standard deviation (SD) or n (%). BMI, body mass index.

**Table 2:** Association analyses of pre-pregnancy BMI (continuous variable) with maternal characteristics.

	β or OR (95%CI)	*p* Value
Maternal age (years) ^1^	0.05 (−0.31, 0.42)	0.773
Height (cm) ^1^	−0.34 (−0.82, 0.14)	0.159
Gestational weight gain (kg/40 weeks) ^1^	−0.14 (−0.44, 0.16)	0.365
Parity (multipara) ^2^	1.18 (0.98, 1.44)	0.094
Maternal educational attainment, university or higher degree ^2^	0.97 (0.80, 1.20)	0.768
Household income (≥6 million yen per year) ^2^	0.86 (0.71, 1.04)	0.113
Gestational week at delivery (week) ^1^	0.01 (−0.11, 0.13)	0.877

^1^ Linear regression analyses and ^2^ logistic regression analyses were performed with pre-pregnancy BMI as the explanatory variable. BMI, body mass index; β, regression coefficient; OR, odds ratio; CI, confidence interval.

**Table 3:** Verification of stability in diet quality classification based on the NRF9.3.

	Diet Quality Classification		
	Low_Period (3-Day) NRF9.3 Tertile T1 *n* = 30	Intermediate_Period (3-Day) NRF9.3 Tertile T2 *n* = 30	High_Period (3-Day) NRF9.3 Tertile T3 *n* = 31
Probability (Low_day) ^a^	0.704 ± 0.256	0.260 ± 0.207	0.034 ± 0.087
Probability (Intermediate_day) ^b^	0.227 ± 0.239	0.540 ± 0.292	0.226 ± 0.203
Probability (High_day) ^c^	0.069 ± 0.120	0.200 ± 0.184	0.740 ± 0.222

Values are represented as mean ± standard deviation (SD). ^a^ Probability of being classified as lowest tertile when one-day dietary record was randomly extracted 1000 times. ^b^ Probability of being classified as intermediate tertile when one-day dietary record was randomly extracted 1000 times. ^c^ Probability of being classified as highest tertile when one-day dietary record was randomly extracted 1000 times.

**Table 4:** Association of pre-pregnancy BMI ^a^ with NRF9.3 and total energy intake.

	Unadjusted		Adjusted ^c^	
	β (95%CI)	*p* Value	β (95%CI)	*p* Value
NRF9.3 score	−258 (−447, −70)	0.0079	−280 (−463, −96)	0.0032
Staple foods (grain dishes) (SV/d)	−0.011 (−1.866, 1.845)	0.991	−0.269 (−2.164, 1.626)	0.778
Side dishes (vegetable dishes) (SV/d)	−0.315 (−3.262, 2.632)	0.832	−0.202 (−3.177, 2.773)	0.893
Main dishes (fish and meat dishes) (SV/d)	0.442 (−2.918, 3.802)	0.794	0.973 (−2.403, 4.348)	0.568
Milk ^b^(SV/d)	1.532 (−0.541, 3.605)	0.146	1.61 (−0.442, 3.662)	0.122
Fruit ^b^(SV/d)	0.864 (−1.436, 3.164)	0.457	0.879 (−1.455, 3.213)	0.456
Energy intake from snacks ^b^	7.7 (−12.6, 28)	0.453	8.8 (−12.2, 29.9)	0.407
Total energy intake	549 (109, 989)	0.015	549 (93, 1005)	0.019
JFGST score	0.596 (−12.521, 13.714)	0.928	−0.646 (−13.731, 12.439)	0.922

^a^ The values were log transformed because of the skewness of the BMI distribution. ^b^ The values were Box–Cox transformed because they were not normally distributed. ^c^ Adjusted for maternal age, educational attainment, and household income. β, regression coefficient; BMI, body mass index; NRF9.3, Nutrient Rich-Food Index 9.3; JFGST, Japanese Food Guide Spinning Top; SV/d, serving numbers per day.

**Table 5:** Relationship between diet quality assessed by NRF9.3 and GWG or birth weight.

	Diet Quality			*p* for Trend ^a^	
	Low_Period NRF9.3 Tertile T1 (*n* = 30)	Intermediate_Period NRF9.3 Tertile T2 (*n* = 30)	High_Period NRF9.3 Tertile T3 (*n* = 31)	Unadjusted	Adjusted
GWG (kg/40 weeks)	11.4 ± 3.1	9.9 ± 3.8	9.3 ± 3.0	0.018	0.009
BWGA Z-scores	0.03 ± 0.83	0.10 ± 1.04	0.02 ± 0.96	0.936	0.851

^a^ Adjusted for maternal age, pre-pregnancy BMI, educational attainment, and household income. GWG, gestational weight; BWGA, birth weight for gestational age.

## Data Availability

The datasets used and/or analyzed during the current study are shown in the Supplementary Materials.

## Supplementary Materials

The following supporting information can be downloaded at: https://www.mdpi.com/article/10.3390/nu15081827/s1, Figure S1: Relationship between pre-pregnancy BMI and estimated physical activity level; Figure S2: Relationship between Japanese Food Guide Spinning Top (JFGST) adherence score and GWG adequacy; Figure S3: Relationship among Nutrient-Rich Food index 9.3 (NRF9.3), Japanese Food Guide Spinning Top (JFGST), adherence score and weight characteristics of pregnant women; Table S1: Calculation method of the Japanese Food Guide Spinning Top adherence score for women during mid-gestation; Table S2: Classification according to gestational weight gain adequacy; Table S3: The Japanese Food Guide Spinning Top component score and total adherence score by pre-pregnancy BMI category; Table S4: Relationship between energy intake and GWG or birth weight.
