# Supplementary material for: Diet Quality and Its Relationship with Weight Characteristics in Pregnant Japanese Women: A Single-Center Birth Cohort Study
Source: Nutrients. 2023 Apr 10;15(8):1827. doi: 10.3390/nu15081827 (PMC10142925; doi:10.3390/nu15081827)
Supplement: Supplementary file 1 [file nutrients-15-01827-s001.zip › SupplementaryFile_Diet_quality_20230328.pptx]

## Slide 1
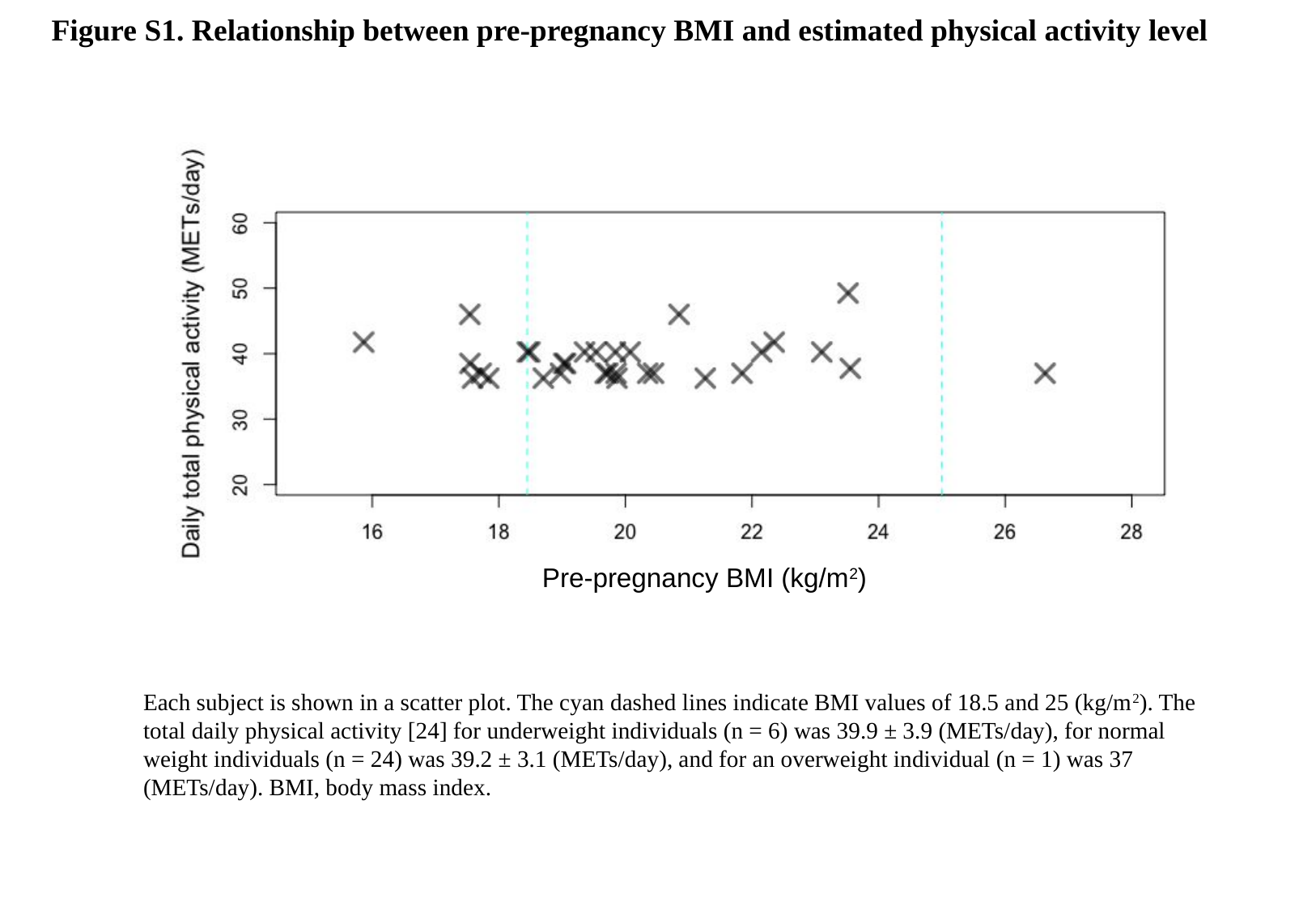

Figure S1. Relationship between pre-pregnancy BMI and estimated physical activity level
Pre-pregnancy BMI (kg/m2)
Each subject is shown in a scatter plot. The cyan dashed lines indicate BMI values of 18.5 and 25 (kg/m2). The total daily physical activity [24] for underweight individuals (n = 6) was 39.9 ± 3.9 (METs/day), for normal weight individuals (n = 24) was 39.2 ± 3.1 (METs/day), and for an overweight individual (n = 1) was 37 (METs/day). BMI, body mass index.

## Slide 2
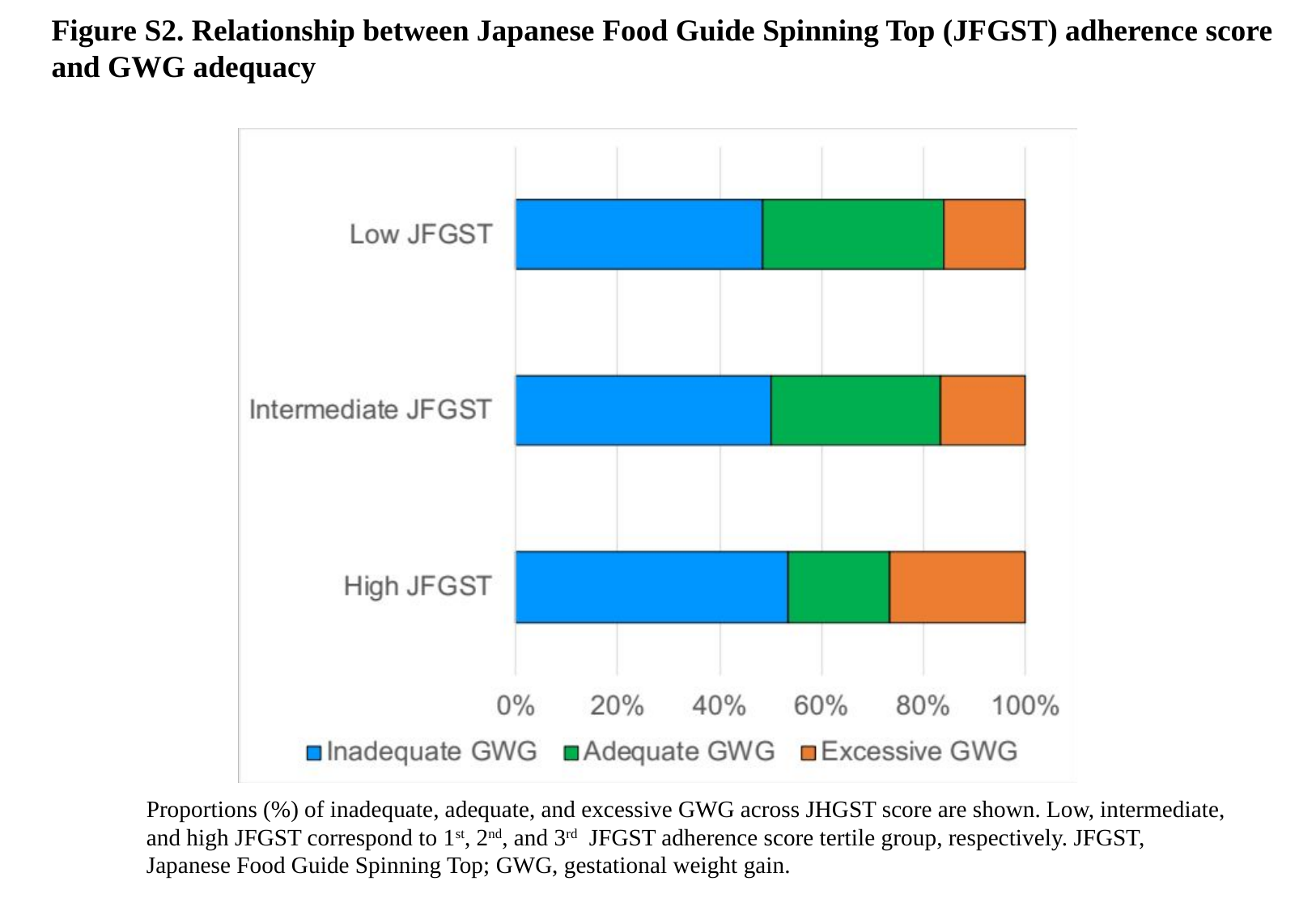

Figure S2. Relationship between Japanese Food Guide Spinning Top (JFGST) adherence score and GWG adequacy
Proportions (%) of inadequate, adequate, and excessive GWG across JHGST score are shown. Low, intermediate, and high JFGST correspond to 1st, 2nd, and 3rd JFGST adherence score tertile group, respectively. JFGST, Japanese Food Guide Spinning Top; GWG, gestational weight gain.

## Slide 3
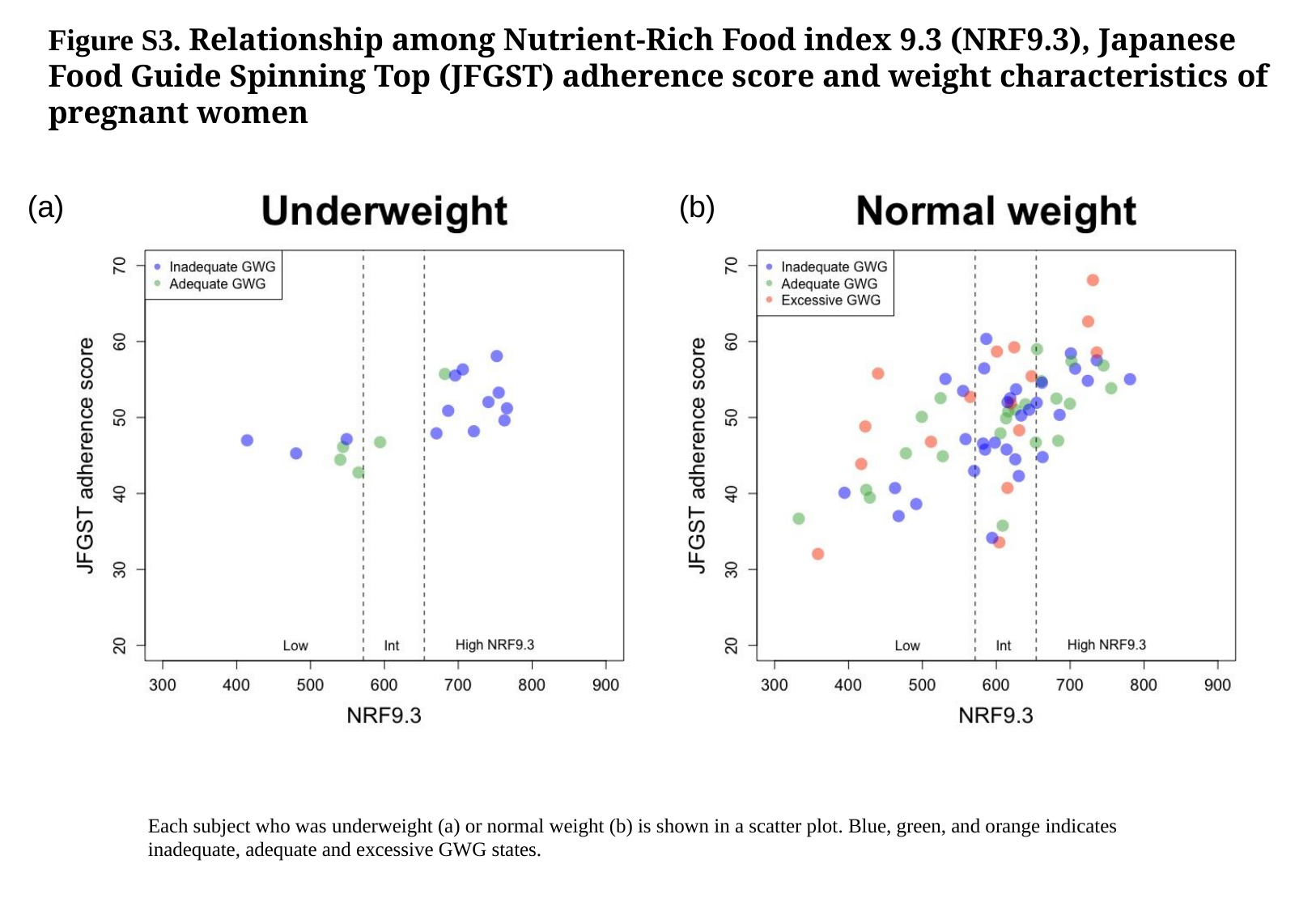

Figure S3. Relationship among Nutrient-Rich Food index 9.3 (NRF9.3), Japanese Food Guide Spinning Top (JFGST) adherence score and weight characteristics of pregnant women
(a)
(b)
Each subject who was underweight (a) or normal weight (b) is shown in a scatter plot. Blue, green, and orange indicates inadequate, adequate and excessive GWG states.

## Slide 4
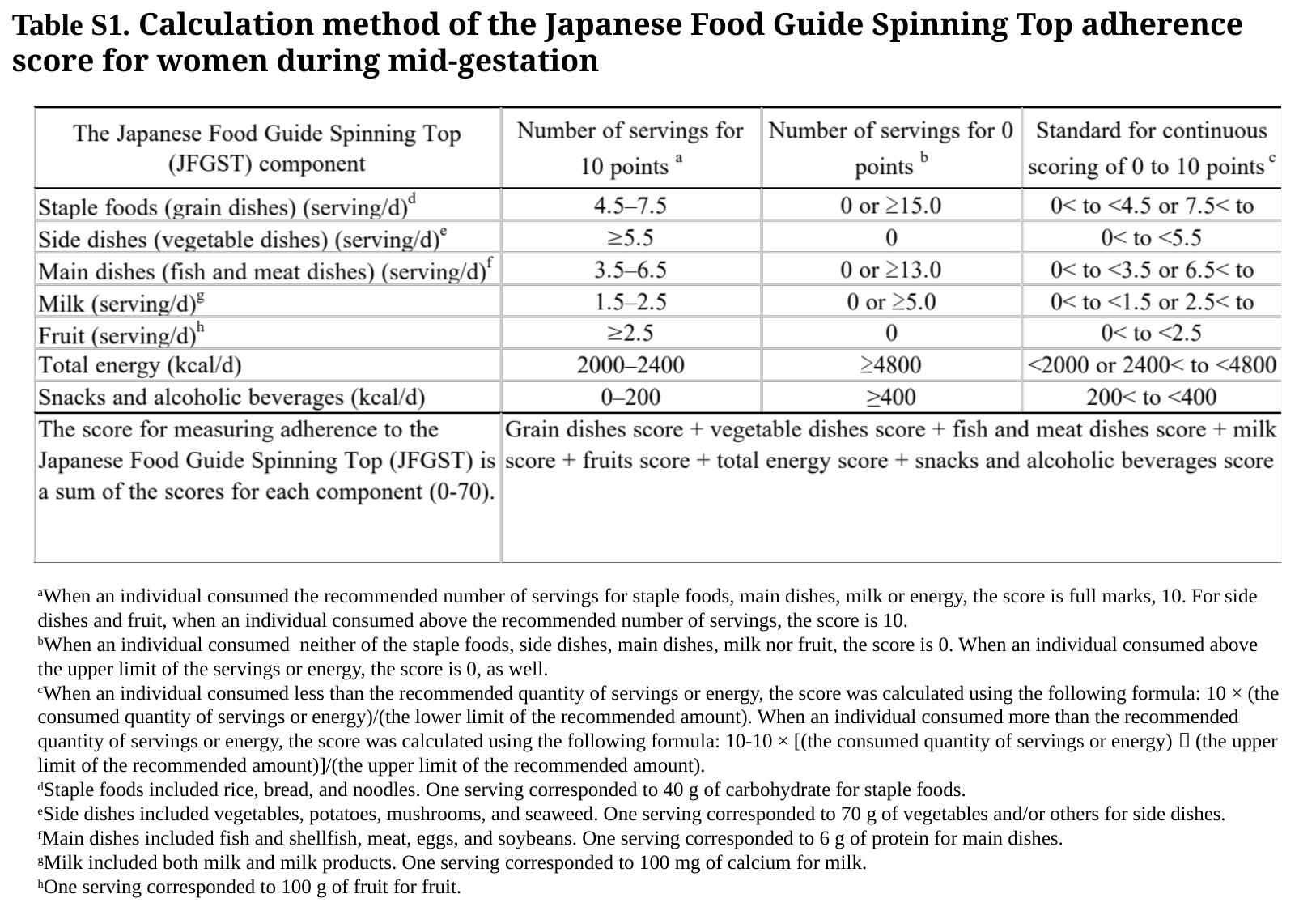

Table S1. Calculation method of the Japanese Food Guide Spinning Top adherence score for women during mid-gestation
aWhen an individual consumed the recommended number of servings for staple foods, main dishes, milk or energy, the score is full marks, 10. For side dishes and fruit, when an individual consumed above the recommended number of servings, the score is 10.
bWhen an individual consumed neither of the staple foods, side dishes, main dishes, milk nor fruit, the score is 0. When an individual consumed above the upper limit of the servings or energy, the score is 0, as well.
cWhen an individual consumed less than the recommended quantity of servings or energy, the score was calculated using the following formula: 10 × (the consumed quantity of servings or energy)/(the lower limit of the recommended amount). When an individual consumed more than the recommended quantity of servings or energy, the score was calculated using the following formula: 10-10 × [(the consumed quantity of servings or energy)－(the upper limit of the recommended amount)]/(the upper limit of the recommended amount).
dStaple foods included rice, bread, and noodles. One serving corresponded to 40 g of carbohydrate for staple foods.
eSide dishes included vegetables, potatoes, mushrooms, and seaweed. One serving corresponded to 70 g of vegetables and/or others for side dishes.
fMain dishes included fish and shellfish, meat, eggs, and soybeans. One serving corresponded to 6 g of protein for main dishes.
gMilk included both milk and milk products. One serving corresponded to 100 mg of calcium for milk.
hOne serving corresponded to 100 g of fruit for fruit.

## Slide 5
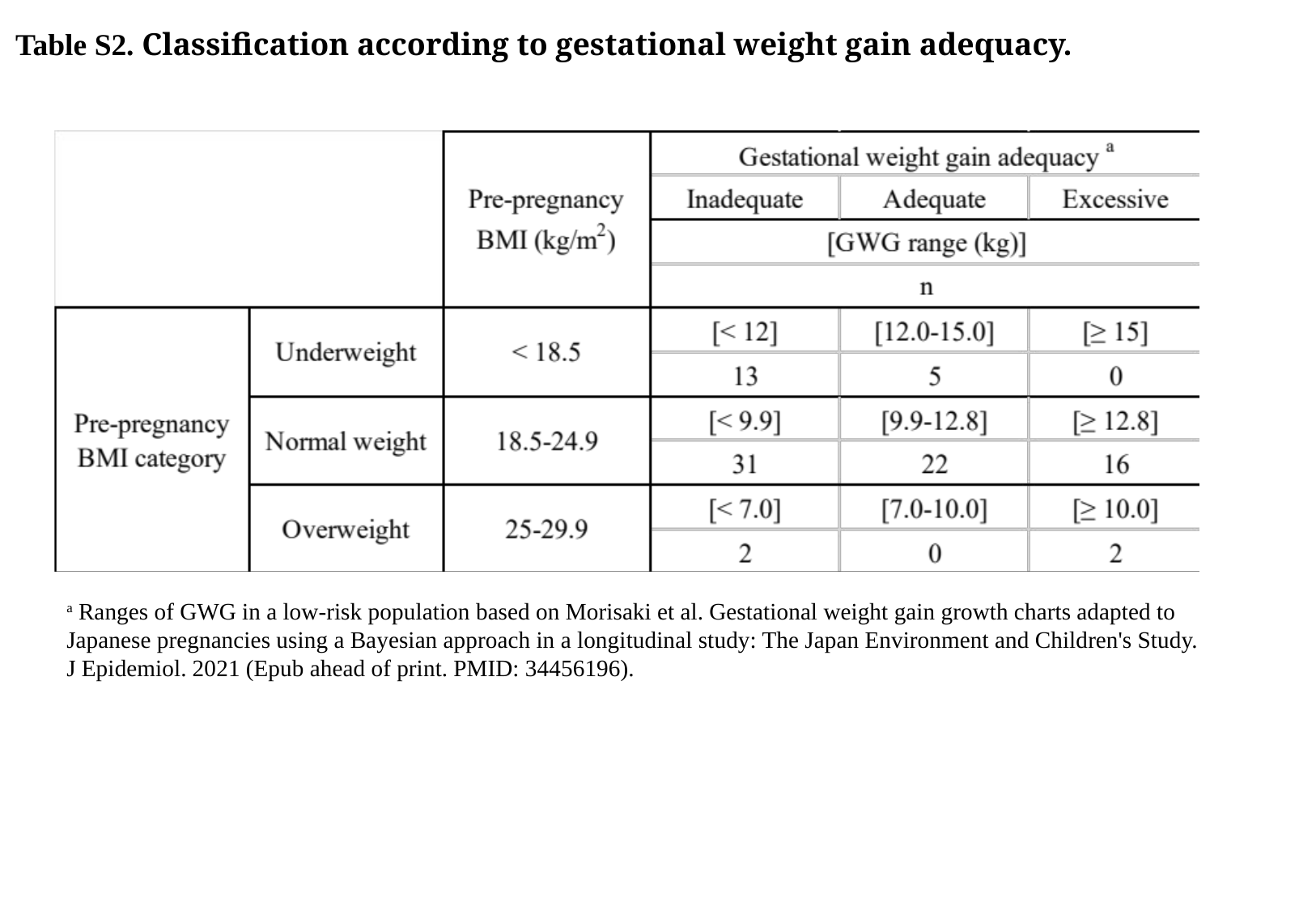

Table S2. Classification according to gestational weight gain adequacy.
a Ranges of GWG in a low-risk population based on Morisaki et al. Gestational weight gain growth charts adapted to Japanese pregnancies using a Bayesian approach in a longitudinal study: The Japan Environment and Children's Study. J Epidemiol. 2021 (Epub ahead of print. PMID: 34456196).

## Slide 6
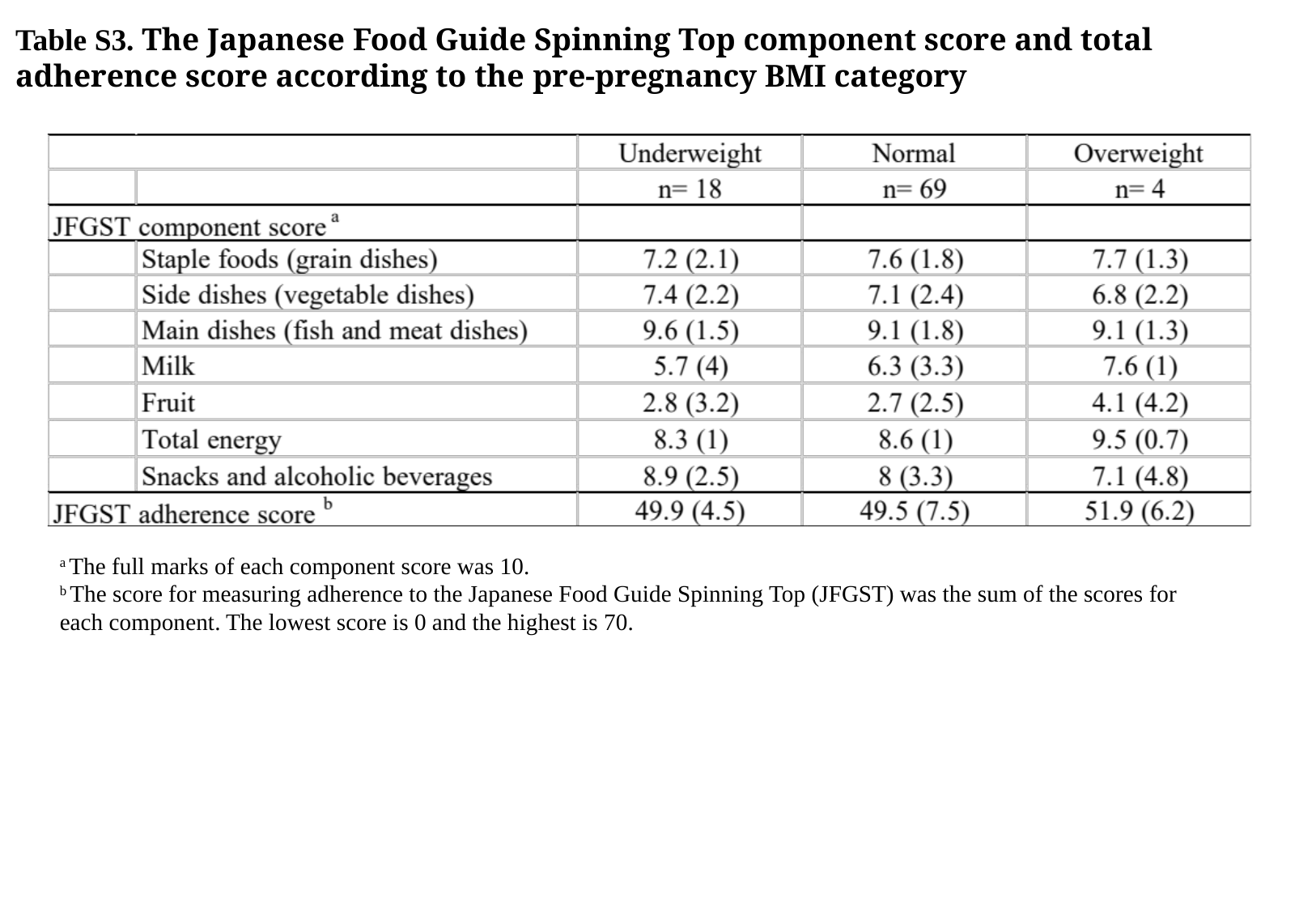

Table S3. The Japanese Food Guide Spinning Top component score and total adherence score according to the pre-pregnancy BMI category
a The full marks of each component score was 10.
b The score for measuring adherence to the Japanese Food Guide Spinning Top (JFGST) was the sum of the scores for each component. The lowest score is 0 and the highest is 70.

## Slide 7
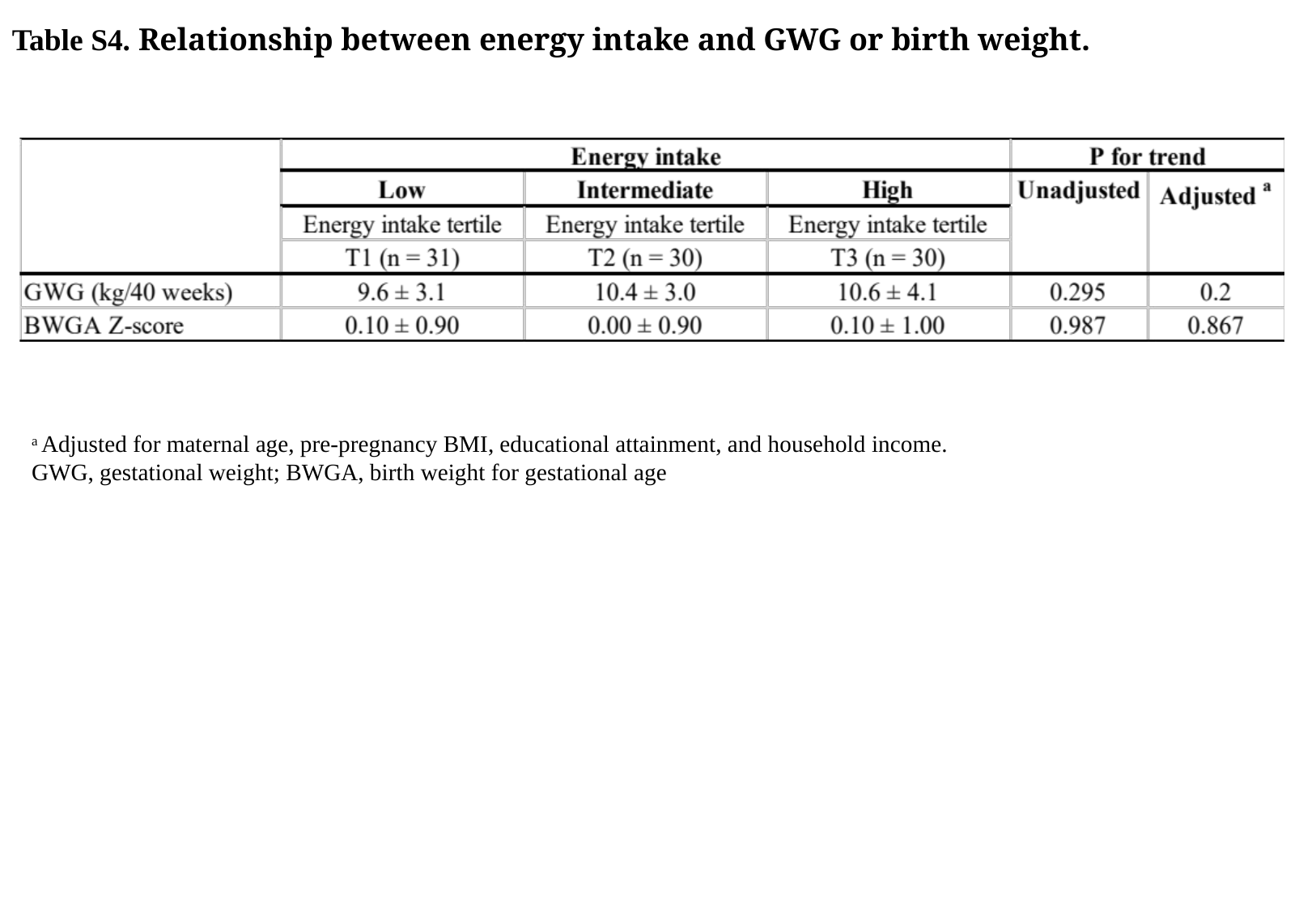

Table S4. Relationship between energy intake and GWG or birth weight.
a Adjusted for maternal age, pre-pregnancy BMI, educational attainment, and household income.
GWG, gestational weight; BWGA, birth weight for gestational age
